# Acetylation and deacetylation of Cdc25A constitutes a novel mechanism for modulating Cdc25A functions with implications for cancer

**DOI:** 10.18632/oncotarget.7966

**Published:** 2016-03-07

**Authors:** Enerlyn M. Lozada, Zdenek Andrysik, Moying Yin, Nicholas Redilla, Kathryn Rice, Peter J. Stambrook

**Affiliations:** ^1^ Department of Molecular Genetics, University of Cincinnati College of Medicine, Cincinnati, Ohio 45267, USA; ^2^ Current affiliation: Department of Pharmacology, University of Colorado Anschutz Medical Campus, Aurora, Colorado 80045, USA

**Keywords:** Cdc25A acetylation, ARD1, HDAC11, DNA damage, cancer

## Abstract

The dual specificity phosphatase Cdc25A is a key regulator of the cell cycle that promotes cell cycle progression by dephosphorylating and activating cyclin-dependent kinases. In response to genotoxicants, Cdc25A undergoes posttranslational modifications which contribute to its proteasome-mediated degradation and consequent cell cycle checkpoint arrest. The most thoroughly studied Cdc25A modification is phosphorylation. We now provide the first evidence that Cdc25A can be acetylated and that it directly interacts with the ARD1 acetyltransferase which acetylates Cdc25A both biochemically and in cultured cells. When acetylated, Cdc25A has an extended half-life. We have also identified the class IV histone deacetylase, HDAC11, as a Cdc25A deacetylase. We further show that DNA damage, such as exposure to methyl methanesulfonate (MMS), etoposide or arsenic, increases Cdc25A acetylation. Importantly, this acetylation modulates Cdc25A phosphatase activity and its function as a cell cycle regulator, and may reflect a cellular response to DNA damage. Since Cdc25A, ARD1, and HDAC11 are frequently dysregulated in multiple types of cancer, our findings may provide insight into a novel mechanism in carcinogenesis.

## INTRODUCTION

The Cdc25 family of phosphatases acts on the Cdk/cyclin complexes to promote transitions between cell cycle phases [1]. In humans there are three Cdc25 family members, Cdc25A, B and C, of which Cdc25A alone is essential for viability during early embryonic development. The absence of Cdc25A results in fetal lethality. Mice that are null for Cdc25A die between embryonic days 5 and 7 [2] whereas mice that are null for Cdc25B or Cdc25C are fully viable [3, 4]. The activity and abundance of Cdc25A are intricately regulated and Cdc25A is frequently overexpressed in several cancer types [5–8]. Phosphorylation and ubiquitination modulate its stability, its interaction with other proteins, and possibly its enzymatic activity [9–16]. Since the acetyltransferase ARD1 was found to interact with Cdc25A in a yeast two-hybrid screen [17], we hypothesized that Cdc25A is also regulated by acetylation and deacetylation.

While most acetylation studies have focused on chromatin-associated proteins, acetylation of non-chromatin proteins has been less thoroughly explored. There are multiple genes that encode acetyltransferases [18–20], including ARD1 of which there are two isoforms: ARD1A and ARD1B [21]. It is believed that ARD1B is a bona fide retrogene of ARD1A [22]. ARD1 acetylates both internal lysine ε-amino groups and N-terminal α- amino groups in mammalian cells [23]. The abnormal of expression of ARD1 has been reported in at least eight cancer types [24–30], while its regulated expression is required for normal human development. Ogden syndrome, which results in early infant death, is a consequence of germline defects in N-terminal acetylation by ARD1 [31].

Under homeostatic conditions, the steady state protein acetylation status is determined by the reciprocal action of acetylases and deacetylases. The deacetylases are grouped into four classes based primarily on their cellular localization and catalytic mechanisms of action [32]. Classes I, II and IV require a zinc molecule in their active site, whereas Class III members, also known as sirtuins, require NAD+ for their deacetylase activity [33]. The most recently identified and least well studied deacetylase is the Class IV member, HDAC11 [34]. Its depletion is sufficient to cause cell death and to inhibit metabolic activity in multiple cancer cell lines [35], suggesting a potential role in cancer cell survival.

The present study is the first to report acetylation as a previously undescribed Cdc25A post-translational modification. We have characterized its acetylation and deacetylation and its biological effects on Cdc25A stability and function. When acetylated, its half-life increases and its phosphatase activity is diminished. We show that Cdc25A acetylation occurs naturally within cells and can be stimulated by treatments that cause DNA damage. Furthermore, we show that ARD1 and HDAC11 each interact directly with Cdc25A *in vitro* and in cultured cells, demonstrating that the antagonistic actions of ARD1 and HDAC11 regulate the level of Cdc25A acetylation. Collectively, our findings show that Cdc25A acetylation is a subtle regulatory mechanism for controlling Cdc25A activity. It is therefore not surprising that Cdc25A and the proteins involved in its acetylation status are aberrantly expressed in several cancer types.

## RESULTS

### Cdc25A and ARD1 interact both *in vivo* and *in vitro*

**Figure 1:**
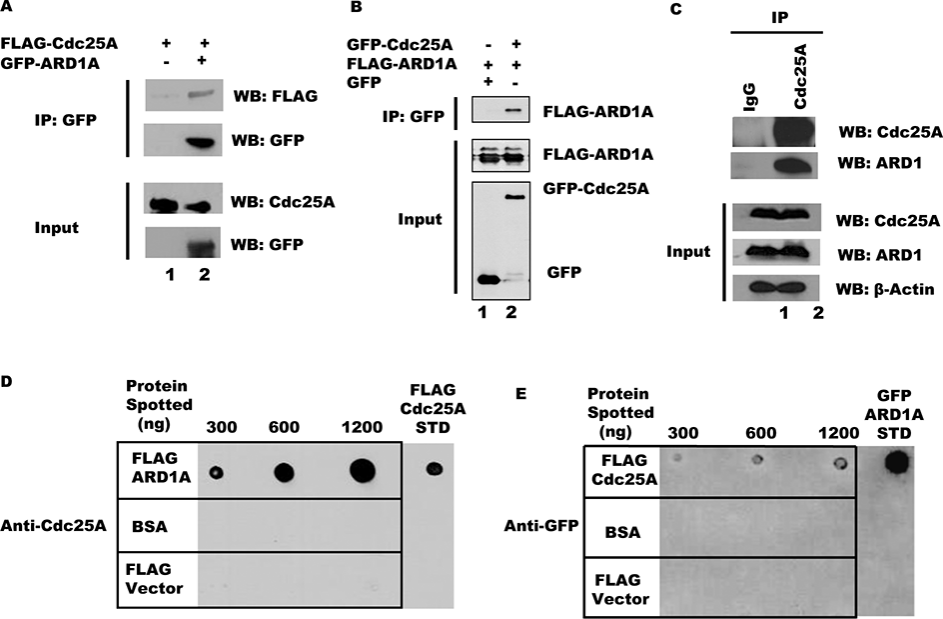
Cdc25A and ARD1 interact *in vivo* and *in vitro* (**A**) Cdc25A co-immunoprecipitates with ARD1 *in vitro*. Purified FLAG-Cdc25A was incubated with anti-GFP beads, either alone or in combination with GFP-ARD1A. Immunoprecipitated products were subjected to SDS-PAGE and probed with anti-GFP and anti-FLAG antibodies. (**B**) ARD1 and Cdc25A associate after co-transfection. FLAG-ARD1A was co-transfected into HEK 293T cells with either GFP-Cdc25A or the GFP vector alone. After 24 hours, cell lysates were subjected to immunoprecipitation using anti-GFP antibody. Precipitated proteins were examined by Western blot. (**C**) Co-immunoprecipitation of Cdc25A and ARD1 in cultured cells. HEK 293T cell lysates were subjected to immunoprecipitation using antibody to Cdc25A or mouse IgG. Immunoprecipitates were analyzed with anti-ARD1 and anti-Cdc25A antibodies. (**D**) and (**E**) Cdc25A-ARD1 interact directly. Purified ARD1A (D), Cdc25A (E), and a BSA control were immobilized on nitrocellulose membranes at the concentrations indicated above each panel. Membranes were incubated in buffer containing (D) Cdc25A (2400 ng) or (E) ARD1A (720 ng). Immunodetection of bound protein was performed with anti-Cdc25A (D) or anti-GFP (E) antibodies.

Since Cdc25A and ARD1 physically interact in a yeast two-hybrid assay [17], we asked whether Cdc25A and ARD1 can interact in a mammalian system. As a first step, purified FLAG-Cdc25A fusion protein was incubated with GFP-ARD1A, and complexes formed *in vitro* were immunoprecipitated with anti-GFP beads. As shown in Figure 1A, FLAG-Cdc25A co-immunoprecipitated with GFP-ARD1A. Similarly, purified GFP-ARD1A co-immunoprecipitated with FLAG-Cdc25A *in vitro* using anti-FLAG M2 beads (Supplementary Figure S1A). To test whether these two proteins can interact within cultured mammalian cells, HEK 293T cells were co-transfected with plasmids expressing GFP-Cdc25A and FLAG-tagged ARD1A. Following Cdc25A immunoprecipitation from cell lysates with anti-GFP antibody, FLAG-ARD1 was detected by Western blot (Figure 1B), suggesting that these two proteins are part of the same intracellular complex. The reciprocal experiment, in which endogenous ARD1 was immunoprecipitated from HEK 293T cell lysates followed by probing for Cdc25A, also supported interaction between these proteins (Supplementary Figure S1B). To test this proposition, endogenous Cdc25A was immunoprecipitated from HEK 293T cell lysates, immunoprecipitates resolved by SDS-PAGE, and probed with an anti-ARD1 antibody. As shown in Figure 1C, endogenous ARD1 protein clearly co-immunoprecipitated with Cdc25A. Since co-immunoprecipitations can reflect direct or indirect interactions, Far Western experiments were performed. ARD1A was spotted onto a membrane matrix in increasing concentration followed by incubation with a constant amount of Cdc25A protein. The relative amount of Cdc25A directly bound to ARD1A was monitored by incubation with antibody to Cdc25A. As shown in Figure 1D, Cdc25A bound to ARD1 in a dose dependent manner. The reciprocal was also true (Figure 1E) indicating that not only are Cdc25A and ARD1 members of the same complex, but that they bind directly to each other.

### ARD1 acetylates Cdc25A *in vitro* and in cells

**Figure 2:**
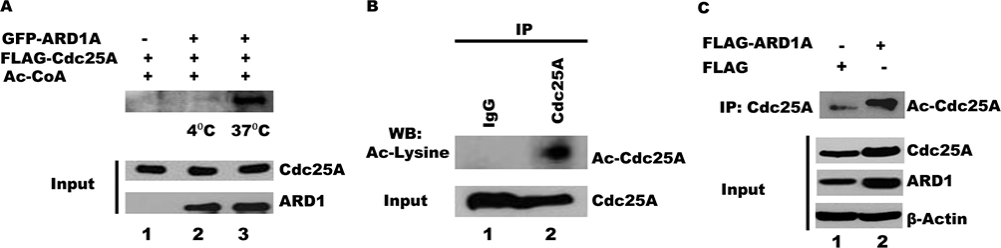
ARD1 acetylates Cdc25A *in vitro* and in cells (**A**) ARD1 mediates Cdc25A acetylation *in vitro*. Purified GFP-ARD1A was incubated at 4°C, or 37°C, with purified FLAG-Cdc25A. The reactions were analyzed using anti-acetyl lysine antibody, followed by chemiluminescent detection. (**B**) Cdc25A is endogenously acetylated. Cdc25A was immunoprecipitated from HEK 293T cell lysates and its acetylation status was analyzed by Western blot using an acetyl-specific antibody. (**C**) ARD1 overexpression increases Cdc25A acetylation in cells. Cdc25A was immunoprecipitated from HEK 293T cell lysates transfected with FLAG-ARD1A and the Cdc25A acetylation status was analyzed by Western blot using an antibody specific for acetylated lysines.

It is known that Cdc25A undergoes extensive posttranslational phosphorylation and ubiquitination [9–16]. Its association with ARD1, an acetyltransferase [36], now suggests that Cdc25A may also be subject to acetylation. To test whether ARD1 can mediate Cdc25A acetylation *in vitro*, purified GFP-ARD1A was incubated with FLAG-Cdc25A in the presence of acetyl CoA. As shown in Figure 2A, ARD1 directly acetylates Cdc25A when the reaction is run at 37°C (lane 3) but not at 4°C (lane 2), indicating that ARD1 alone, independent of other cellular components, is sufficient to carry out this reaction. Whether Cdc25A undergoes acetylation *in vivo* has not been reported. To assess the acetylation status of Cdc25A in cultured cells, Cdc25A was immunoprecipitated from HEK 293T cell lysates, separated by gel electrophoresis and challenged with antibody to acetyl lysine. We show for the first time, that some endogenous Cdc25A exists in an acetylated form (Figure 2B). If ARD1 is an acetyltransferase that acetylates Cdc25A, one would predict that elevated levels of ARD1 would result in more Cdc25A acetylation. To this end, ARD1 was overexpressed in HEK 293T cells by transfection with a plasmid encoding FLAG-ARD1A or FLAG alone as a control. Following immunoprecipitation of Cdc25A and separation by SDS PAGE, the blots were challenged with anti-acetyl lysine antibody. Figure 2C (compare lanes 1 and 2) clearly shows that the Cdc25A acetylation level is increased in cells that overexpress ARD1, supporting the contention that Cdc25A is a substrate for acetylation by ARD1.

### ARD1 expression affects Cdc25A protein stability by modulating its ubiquitination

**Figure 3:**
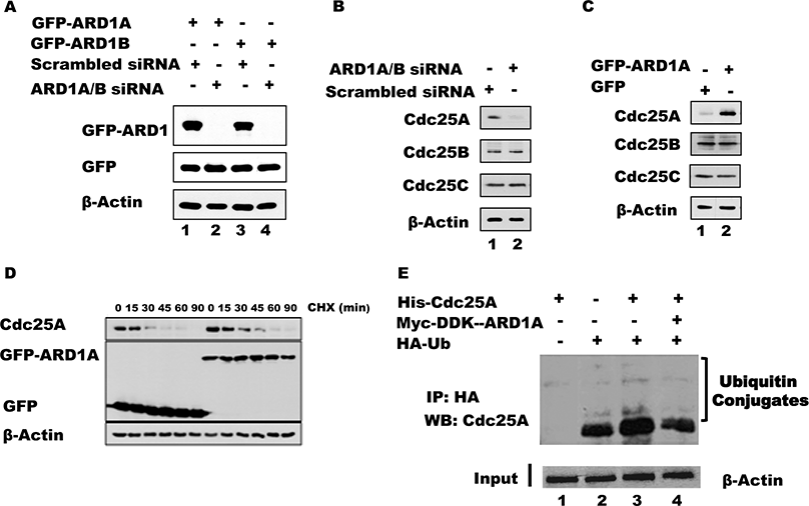
ARD1 expression affects Cdc25A protein stability by modulating its ubiquitination (**A**) ARD1 knockdown efficiency. HEK 293 cells were transfected with siRNA against ARD1A/B or with scrambled siRNA as control to establish ARD1 knockdown. (**B**) ARD1A/B knockdown induces a reduction in Cdc25A level, but not of Cdc25B or Cdc25C. (**C**) Overexpression of ARD1 in HEK 293 cells increases the level of Cdc25A but not of Cdc25B, and Cdc25C. (**D**) ARD1 increases Cdc25A half-life. HEK 293 cells were transfected with GFP vector of GFP-ARD1A followed by treatment of cells with CHX for the indicated time. Cell lysates were analyzed by Western Blot with anti-Cdc25A, anti-GFP, and anti-β-actin antibodies. (**E**) ARD1 decreases Cdc25A ubiquitination. Cell lysates from HEK 293T were incubated with HA-Ub, His-Cdc25A, and/or Myc-DDK-ARD1. After immunoprecipitation with an anti-HA antibody, the immonoprecipitates were subjected to Western blot analysis using an anti-Cdc25A antibody.

In addition to elevated levels of acetylated Cdc25A following ARD1 overexpression, the absolute level of Cdc25A is increased (Figure 2C). To further address whether ARD1 can affect the level of Cdc25A protein, ARD1 was overexpressed or depleted from HEK 293T cells using siRNAs against ARD1, and the levels of Cdc25A were assessed by immunoblotting. Efficient knock-down of both ARD1A and ARD1B was achieved with a combination of two different siRNAs, referred to as ARD1A/B siRNA (Figure 3A). Depletion of ARD1 mediated by siRNA reduced the level of endogenous Cdc25A but had no effect on the levels of Cdc25B and Cdc25C (Figure 3B). Conversely, ectopic expression of GFP-ARD1A increased the abundance of Cdc25A (Figure 3C). Again, there was no significant effect on the levels of either Cdc25B or Cdc25C. Similar results were obtained with ARD1B (data not shown). Collectively, the data suggest a direct role for ARD1 in regulating the abundance of Cdc25A.

To explore whether ARD1 modulates the level of Cdc25A mRNA, and thereby the level of protein, we analyzed Cdc25A mRNA transcript levels by RT-PCR in cells where ARD1 was either depleted or overexpressed (See Supplementary Figure S2 for primers and oligonucleotide sequences). There were no significant changes in mRNA levels (Supplementary Figure S3), suggesting that the observed effect of ARD1 on Cdc25A protein abundance occurs at the post-translational level. These data are consistent with the direct interaction of these proteins and the modification of Cdc25A by ARD1-mediated acetylation. To ask whether elevated ARD1 can stabilize Cdc25A by extending its half-life, which is normally about 15 minutes [1, 37, 38], cells were transfected with GFP-ARD1A or GFP vector alone. Following transfection, the cells were treated with cyclohexamide (CHX) to inhibit protein synthesis and sampled at increasing times to assess the relative abundance of endogenous Cdc25A by immunoblotting. When cells were transfected with GFP-ARD1A the half-life of Cdc25A was increased compared to its half-life in cells transfected with GFP alone. These data suggest that the increased stability of Cdc25A was due to an extended half-life conferred by ARD1-mediated acetylation. It is also noteworthy that the stability of other proteins involved in cell cycle regulation was affected, at least partially, by ARD1A/B overexpression or depletion (Supplementary Figure S4), including cyclin A2, cyclin D1, and the phosphorylated form of retinoblastoma protein (pRB), suggesting a possible broader role for acetylation in cell cycle regulation.

Since Cdc25A stability is regulated by the ubiquitination pathway [1, 39], we asked whether acetylation of Cdc25A and its stabilization by ARD1 is accompanied by a reduction in its ubiquitination. Cell lysates from HEK 293T were incubated with purified Cdc25A and HA-tagged ubiquitin (HA-UB) or with Cdc25A, HA-UB plus ARD1A. After immunoprecipitation with an anti-HA antibody, the immunoprecipitated products were probed by Western blot using an anti-Cdc25A antibody. Figure 3E shows that endogenous Cdc25A is ubiquitinated (lane 2) and transfected Cdc25A is also well ubiquitinated (lane 3). However, the inclusion of ARD1 in the incubation reduced the level of Cdc25A ubiquitination (Figure 3E, lane 4), suggesting that ARD1 stabilizes Cdc25A by modulating the extent of Cdc25A ubiquitination. To test whether cells lacking ARD1 have an increased proteasome-mediated degradation of Cdc25A, proteasome activity was inhibited with MG132. In the presence of MG132, cells transfected with either siRNA against ARD1A/B or with scrambled siRNA displayed strong accumulation of Cdc25A protein. Significantly, when ARD1A/B was depleted the level of Cdc25A accumulation was not as extensive as in control cells (Supplementary Figure S5A), suggesting that other mechanisms influencing Cdc25A stabilization are operative. To further support of our finding that overexpression of ARD1 stabilizes Cdc25A and promotes its accumulation, we showed that cells transfected with GFP-ARD1A had significantly elevated levels of Cdc25A by immunofluorescence compared with cells transfected with GFP alone (Supplementary Figure S5B). These data, which also show co-localization of endogenous Cdc25A with ectopic GFP-ARD1A, are fully consistent with the molecular finding that ARD1 stabilizes Cdc25A.

### DNA damage increases endogenous Cdc25A acetylation

**Figure 4:**
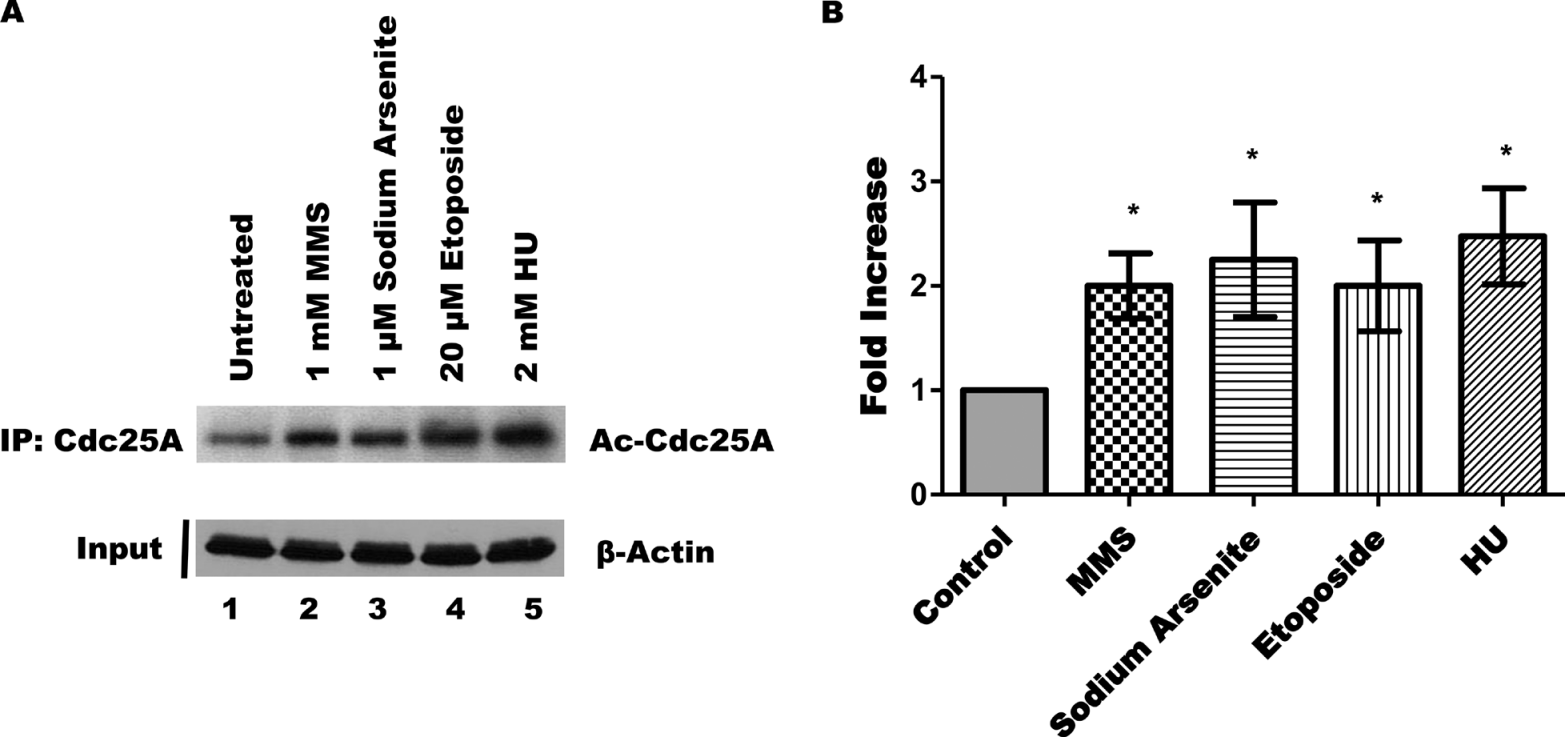
DNA damage increases endogenous Cdc25A acetylation (**A**) HEK 293T cells were treated with MMS, sodium arsenite, etoposide, or HU as described, or left untreated. The acetylation status of Cd25A was assessed by its immunoprecipitation followed by western blot using an acetyl-specific antibody. (**B**) Bar graph from data of Cdc25A acetylation from Panel A (mean ± SEM of 4 independent experiments; *p < 0.05 when compared with untreated cells).

Following DNA damage, several proteins undergo posttranslational modifications that can alter functional activity and subcellular localization [40–42]. To elucidate if DNA damage alters the endogenous Cdc25A acetylation status, we analyzed Cdc25A acetylation levels after treatment with well-known DNA damaging agents. Specifically, HEK 293T cells were treated with methyl methanesulfonate (MMS, an alkylating agent), sodium arsenite (an environmental toxicant), etoposide (a type II topoisomerase poison), or with hydroxyurea (HU, a ribonucleotide reductase inhibitor). When cells were treated with each of the DNA damaging agents, the level of Cdc25A acetylation was increased (Figure 4A, lanes 2–5), indicating that a variety of challenges to DNA integrity significantly enhance the level of Cdc25A acetylation (Figure 4B).

In somatic cells, Cdc25A is degraded in response to DNA damage [43]. We therefore tested whether ectopic expression of GFP-ARD1A interferes with DNA damage-induced Cdc25A degradation. Remarkably, endogenous Cdc25A downregulation induced by etoposide treatment was not prevented by GFP-ARD1A overexpression (Supplementary Figure S6A). However, reduction in the level of Cdc25A was less severe when cells were transfected with ARD1 than when they were not (Supplementary Figure S6A, lane 2 vs. 4). We next transfected cells with a Cdc25A-S82A mutant that is refractory to DNA damage-mediated degradation (Supplementary Figure S6B, see lanes 5 and 6, used as control) [39, 44], to ask whether depletion of ARD1, hence less acetylation, will allow some mutant Cdc25A degradation after etoposide administration. Surprisingly, ARD1A/B depletion led to reduced levels of wild-type and mutant Cdc25A-S82A (Supplementary Figure S6B, compare lane 1 vs. 3, and 5 vs. 7). Taken together, these findings indicate that the ARD1-mediated regulation of Cdc25A stability and the DNA damage-mediated degradation of Cdc25A are two separate processes.

### Cdc25A acetylation modulates its phosphatase activity

**Figure 5:**
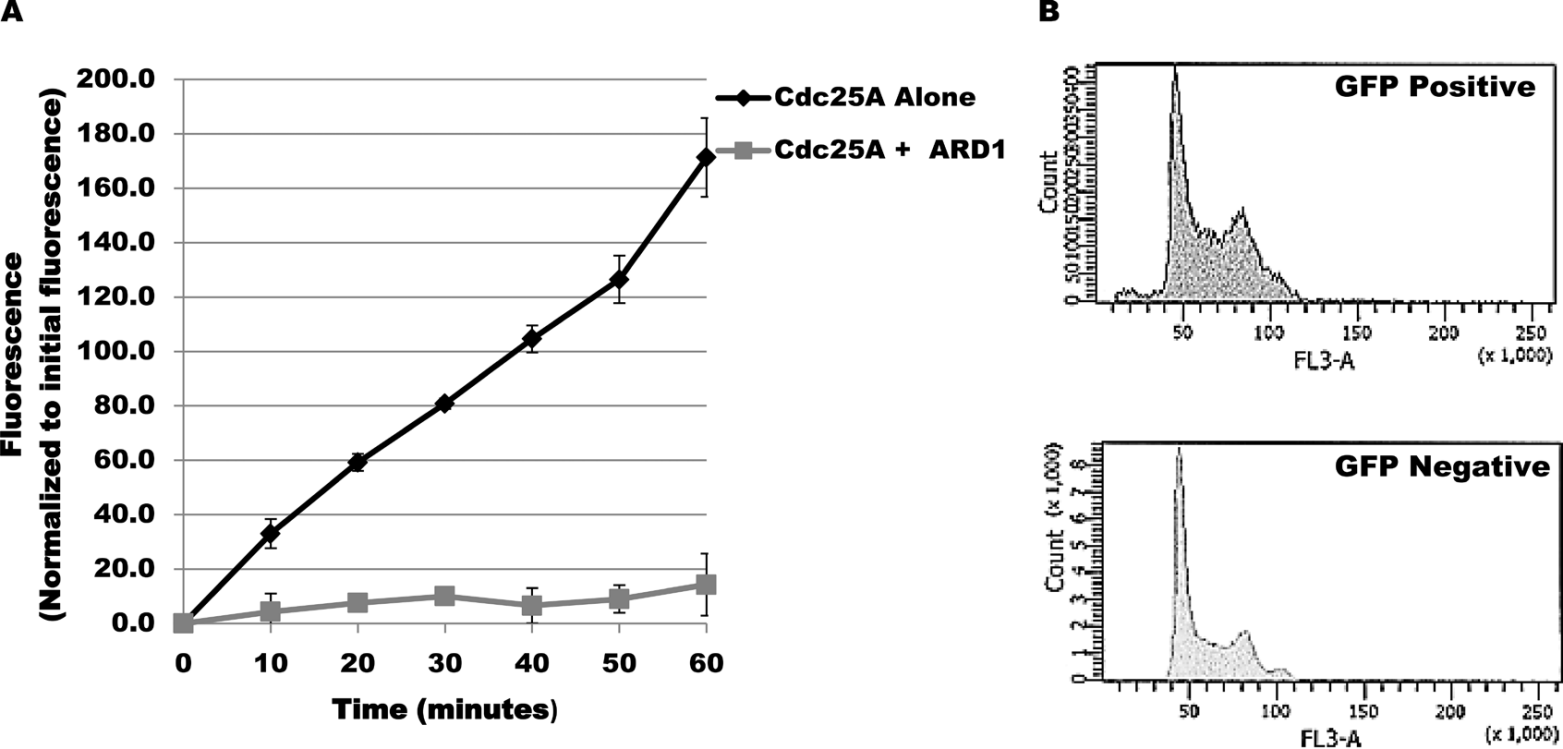
Cdc25A acetylation modulates its phosphatase activity (**A**) ARD1 induces a reduction in Cdc25A phosphatase activity. Phosphatase activity was measured spectrophotometrically based on Cdc25A catalysis of 6, 8-difluoro-4-methylumbelliferyl phosphate (DiFMUP) hydrolysis to 6, 8-difluoro-4-methylumbelliferone. Cdc25A phosphatase activity was measured in the absence or presence of ARD1. (**B**) Cdc25A acetylation is associated with accumulation of cells in the S and G2/M phases of the cell cycle. Cell cycle profiles of untransfected cells (GFP negative) or cells overexpressing GFP-ARD1A (GFP Positive) were compared by flow cytometry.

The change in acetylation level of Cdc25A in response to challenge by genotoxic agents suggests that Cdc25A acetylation might modulate its functional response to genotoxic stress. To ask whether acetylation affects Cdc25A enzymatic activity, purified Cdc25A was incubated alone or with ARD1 in the presence of acetyl-CoA and a phosphorylated substrate that fluoresces when dephosphorylated. As shown in Figure 2A, Cdc25A is acetylated under the conditions used. When assayed at 10 minute intervals during the incubation period, there was a linear increase in fluorescence when ARD1 was omitted from the incubation mix but not when ARD1 was present (Figure 5A), indicating that when Cdc25A is acetylated its phosphatase activity is diminished. Reduction of Cd25A phosphatase activity predicts a disruption in cell cycle regulation. To test this proposition, the cell cycle profile of untransfected cells was compared with that of cells transfected with GFP-ARD1A which results in elevated Cdc25A acetylation. Untransfected cells (GFP negative) showed a normal asynchronous cell cycle profile. In contrast, GFP-ARD1A overexpression produced an altered cell cycle profile with accumulation of cells in S and G2/M (Figure 5B), consistent with increased Cdc25A acetylation and reduction in Cdc25A phosphatase activity as well as disruption of cell cycle regulation.

### Influence of deacetylases, HDACs and sirtuins, on maintaining steady state Cdc25A acetylation levels

**Figure 6:**
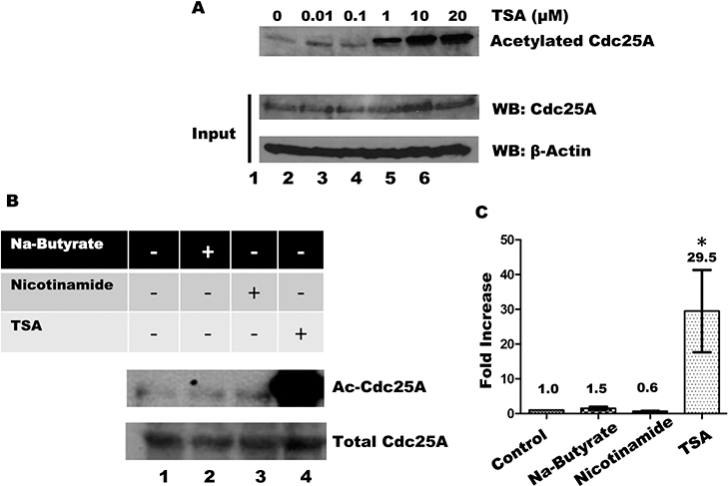
Influence of HDACs and Sirtuins in Cdc25A acetylation levels (**A**) HEK 293T cells were incubated with increasing concentrations of TSA for 4 h. Cdc25A was immunoprecipitated from cell lysates and the immunoprecipitates were subjected to SDS-PAGE and western blotting with anti-acetyl lysine antibody (upper panel). Each cell lysates (100 μg) was analyzed by Western blotting with anti-Cdc25A antibody. (**B**) HEK 293T cells were treated with or without 10 mM Sodium-Butyrate, 5 mM Nicotinamide, and/or 10 μM TSA for 4 h. Cell lysates were subjected to immunoprecipitation with antibody to Cdc25A and immunoprecipitates were subjected to SDS-PAGE and western blotting with anti-acetylated lysine antibody. (**C**) Quantification for Cdc25A acetylation (mean ± SD of 3 independent experiments. * = *P* < 0.05 when compared with control untreated cells).

The intracellular level of protein acetylation is determined by the opposing action of acetyltransferases and deacetylases [45]. Deacetylases are grouped into four classes, I-IV, based on phylogenetic relationships and sequence homology to yeast prototypes [46]. We have used a series of pharmacological inhibitors with overlapping specificities for each of the groups to help establish the deacetylase that acts on Cdc25A. Trichostatin A (TSA) inhibits classes I, II and IV deacetylases, which include most of the deacetylases [47–49]. When cells were treated with increasing concentrations of TSA, Cdc25A acetylation increased in a dose-dependent manner (Figure 6A). To eliminate deacetylases by class, we treated cells with inhibitors specific to different groupings. In addition to TSA, cells were treated with nicotinamide which inhibits Class III deacetylases [50] and sodium butyrate which inhibits Classes I and II deacetylases [48, 51]. As shown in Figure 6B and 6C, only treatment of cells with TSA, but not with nicotinamide or sodium butyrate, resulted in increased Cdc25A acetylation, restricting candidate deacetylases to the Class IV category.

### Cdc25A interacts with HDAC11 *in vivo* and *in vitro*

**Figure 7:**
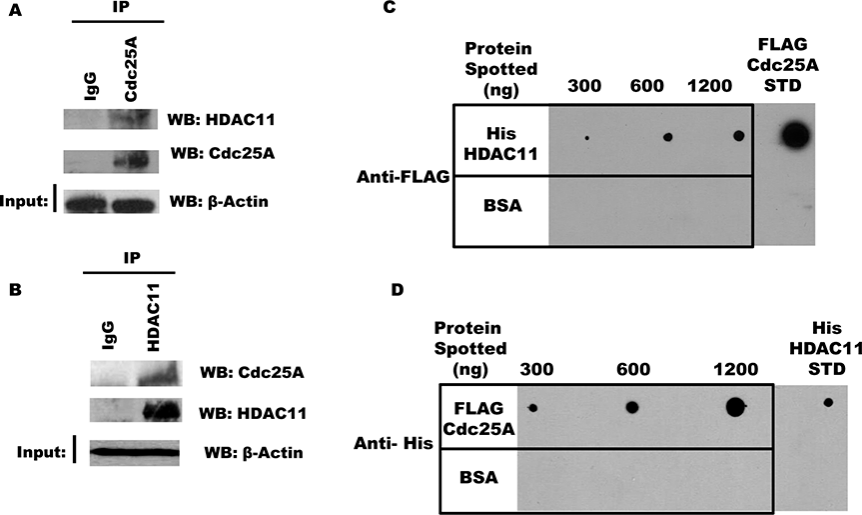
Cdc25A interacts directly with HDAC11 (**A**) and (**B**) Interaction between endogenous Cdc25A and HDAC11. Cdc25A (A) or HDAC11 (B) was immunoprecipitated from HEK 293T cell lysates. Immunoprecipitates were subjected to SDS-PAGE and probed with anti-HDAC11 and anti-Cdc25A antibodies. (**C**) Cdc25A binds directly to HDAC11. Purified His-HDAC11 and BSA (control) were immobilized on nitrocellulose membrane at the indicated concentrations and subsequently incubated in buffer containing FLAG-Cdc25A (800 ng). Cdc25A bound to His-HDAC11 was detected using anti-FLAG antibodies. (**D**) HDAC11 binds directly to Cdc25A. The reciprocal experiment used purified FLAG-Cdc25A and BSA immobilized on a nitrocellulose membrane which was incubated in buffer containing His-HDAC11 (800 ng). Bound His-HDAC11 was detected using anti-His antibodies.

While the Classes I through III deacetylases have several members, Class IV has just one known poorly studied member, HDAC11 [34, 35]. To investigate if Cdc25A and HDAC11 are part of the same endogenous intracellular complex, endogenous Cdc25A was immunoprecipitated from HEK 293T cell lysates with antibody to Cdc25A, or normal IgG as a control, and immunoprecipitates were probed with antibody to HDAC11 by Western blot. As seen in Figure 7A, the two proteins co-immunoprecipitate. Importantly, the interaction is not DNA mediated, as we used buffers containing DNase I during lysis and immunoprecipitation and ethidium bromide during washing of the immunoprecipitate. The reciprocal immunoprecipitation was confirmatory as HDAC11 pulled down Cdc25A (Figure 7B).

Since the above experiments do not exclude indirect association mediated by a bridging or scaffold protein, we used a Far Western approach to ask whether Cdc25A and HDAC11 can interact directly. In one experiment, increasing concentrations of His-HDAC11 were spotted on a membrane matrix, incubated with a solution containing excess FLAG-Cdc25A, followed by probing with antibody to FLAG. As seen in Figure 7C, Cdc25A binding was HDAC11 dose dependent. The reciprocal experiment in which increasing amounts of FLAG-Cdc25A were spotted on the membrane followed by probing for His-HDAC11 yielded similar results (Figure 7D), indicative in both cases of direct interaction between the two proteins.

### Cdc25A is a substrate for HDAC11 deacetylation activity

**Figure 8:**
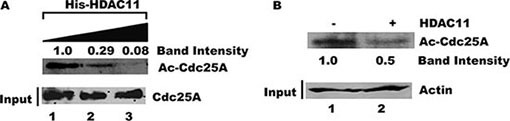
Acetylated Cdc25A is a substrate for deacetylation by HDAC11 (**A**) HDAC11 mediates Cdc25A deacetylation *in vitro*. Purified His-Cdc25A was incubated with increasing amounts of purified HDAC11. The reactions were analyzed using antibody to acetylated lysine followed by chemiluminescent detection. (**B**) Endogenous Cdc25A is deacetylated by HDAC11. Cdc25A was immunoprecipitated from HEK 293T cell lysates and then incubated with or without HDAC11. The enzyme's acetylation status was analyzed by western blot using an acetyl-specific antibody.

Given the close association between Cdc25A and HDAC11, we asked whether Cdc25A might be a substrate for HDAC11 deacetylase activity. Purified Cdc25A was incubated with increasing amounts of His-HDAC11 *in vitro* and the relative acetylation status of Cdc25A was assessed by probing with an anti-acetyl lysine antibody after separation by gel electrophoresis. Figure 8A shows that the amount of acetylated Cdc25A, but not the absolute amount of Cdc25A, decreased with increasing His-HDAC11 concentration. To confirm the HDAC11-mediated deacetylation of endogenous Cdc25A, Cdc25A was immunoprecipitated from HEK 293T cell lysates and incubated with or without HDAC11. Western blots using an anti-acetyl lysine antibody showed that the level of acetylated Cdc25A was reduced following incubation with HDAC11 (Figure 8B), confirming that Cdc25A is a substrate for HDAC11 deacetylation.

## DISCUSSION

We now show for the first time that Cdc25A is acetylated in cells and that acetylation is mediated by the ARD1 acetyltransferase that possesses both N-terminal α-peptide and internal lysine ε-amino acetylation activities [23]. Cdc25A is an unlikely candidate for N-terminal acetylation since its N-terminal residue is glutamic acid [52]. The preferred N-terminal residues for ARD1 acetyltransferase activity are alanine and serine followed by glycine, threonine, valine, and cysteine [53, 54]. Although there are also no published data in support of acetylated Cdc25A, *in silico* software predictors for protein acetylation, such as the Acetylation Set Enrichment-Based (ASEB) program [55] and the lysine acetylation prediction system, LAceP [56], predict acetylation of Cdc25A at several internal lysyl ε-amino groups within the protein. The *in silico* predictions of internal acetylation are consistent with our demonstration of interaction between Cdc25A and ARD1.

When cells are exposed to DNA damaging agents their level of Cdc25A acetylation increases significantly. In addition, we have shown that ARD1 and HDAC11 are the acetyltransferase and deacetylase, respectively, which participate in regulating Cdc25A acetylation status. Both ARD1 and HDAC11 co-immunoprecipitate with Cdc25A from whole cell lysates and also interact directly as purified proteins *in vitro*. The elevated level of Cdc25A acetylation in response to DNA damaging agents suggests that ARD1 and HDAC11 play an important role in the cellular response to genomic insult. The data suggest a multilayered mechanism for maintaining the optimal level of Cdc25A under varying cell physiological conditions. Just as the deubiquitinating enzyme, Dub3, appears to play an antagonizing role to the E3 ubiquitin ligases that promote Cdc25A degradation [57], it is likely that ARD1 plays a similar role to counter excessive degradation.

In addition to protein stabilization, Cdc25A acetylation decreases its phosphatase activity as an additional subtle way to control its functional activity. When we asked whether ARD1-mediated changes in Cdc25A levels were unique to Cdc25A, we found that loss of ARD1 reduced the level of cyclin D1, as well as some other cyclins (Supplementary Figure S4), as previously reported [25]. The changes in the phosphorylated form of pRB are also in agreement with the changes in Cdc25A phosphatase activity, since reduced Cdc25A phosphatase activity should affect the stability and activation of cyclin D-Cdk4 and cyclin A-Cdk2 complexes by preventing phosphorylation of pRB [58–60] as we observe (Supplementary Figure S4).

While expression of Cdc25A [5–8], ARD1 [24–30], and HDAC11 [35] is deregulated in several types of cancers, the mechanism underlying this dysregulation remains unclear. The endogenous interaction between Cdc25A, ARD1, and HDAC11 offers a possible explanation for Cdc25A overexpression based on post-translational stabilization. Since upregulation of ARD1 leads to an elevated Cdc25A level, the Cdc25A-ARD1 and Cdc25A-HDAC11 interactions and their mechanistic and therapeutic implications should be investigated. For example, stabilization of Cdc25A by the acetylation pathway may be responsible, in part, for a subset of cancers where ARD1 is overexpressed or HDAC11 is mutant resulting in elevated levels of Cdc25A.

**Figure 9:**
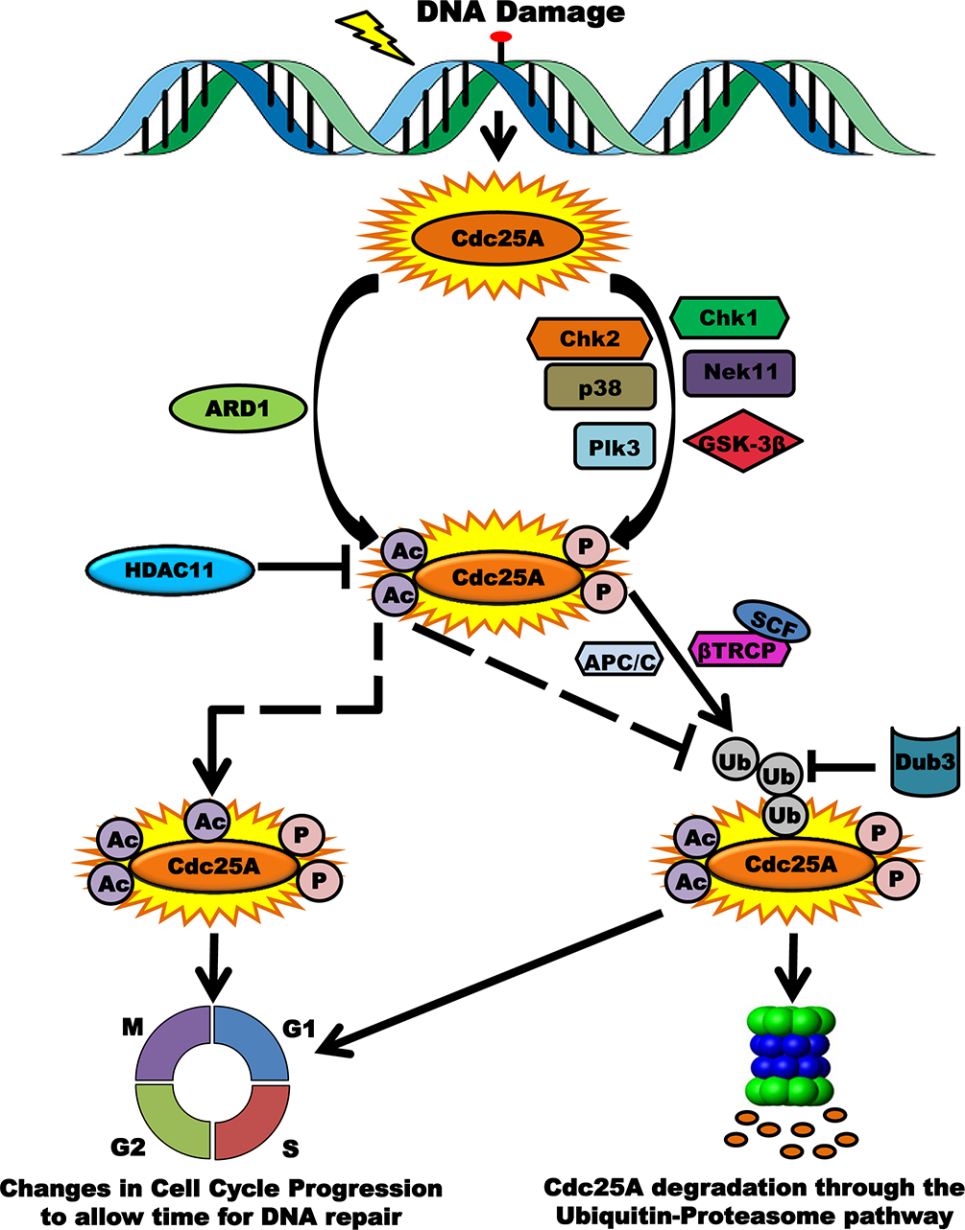
Acetylation as a novel regulatory layer for modulating Cdc25A activity and cell cycle control Following DNA damage, Cdc25A becomes phosphorylated by multiple kinases in preparation for proteasome-mediated degradation. Consistent with data in this report, Cdc25A is also acetylated by ARD1, which is antagonistic to its ubiquitination and degradation. When cells are not challenged, the homeostatic acetylation status of Cdc25A is maintained by the HDAC11 deacetylase. Phosphorylation of Cdc25A promotes its association with the SCF^βTrCP^ E3 ubiquitin ligase, its ubiquitination and subsequent degradation. Acetylation may compete for the same residue as that targeted for ubiquitination (dashed arrow), thereby ameliorating the extent of Cdc25A degradation. Alternatively, Acetylation may occur at distant sites and inhibit ubiquitination via steric interference or conformational changes (elbow dashed arrow). In either case, the consequence of posttranslational modification is disruption of the cell cycle.

This study provides new and revealing results that support the importance of Cdc25A acetylation in response to genotoxic stress and in cancer. We have uncovered a novel regulatory layer for modulating Cdc25A abundance, activity and cell cycle control. Our data are consistent with previous reports implicating Cdc25A in the cellular response to genomic insult, including deregulation of cell cycle progression. We propose a model for how Cdc25A function may be regulated by the acetylation pathway (Figure 9). Under normal cellular homeostasis the level of Cdc25A acetylation is maintained at a steady state through the opposing actions of ARD1 and HDAC11. In response to DNA damage, this balance is perturbed and Cdc25A becomes hyperacetylated and less amenable to degradation. This post-translational modification occurs in addition to phosphorylation by multiple kinases that promote Cdc25A degradation [10, 15, 16, 61, 62]. These modifications, some of which have antagonistic activities, serve as modulators of Cdc25A cellular functions. Phosphorylation prepares Cdc25A for ubiquitination by SCF^βTrCP^, one of the E3-ubiquitin ligases of Cdc25A, the other ubiquitin ligase being APC/Cdh1. This ubiquitination pathway leading to degradation is counteracted by Dub3, which removes ubiquitin conjugates from Cdc25A [57]. In contrast to phosphorylation events that induce degradation, acetylation augments Cdc25A stability. There are at least three scenarios that may explain the mechanism underlying this level of regulation. In one case, there may be competition between acetylation and ubiquitination for modification of the same residue as is the case of Smad7 where ubiquitination and acetylation compete for common lysine residues to control its stability [63]. In the second case, acetylation may modify different residues yet still modulate Cdc25A deubiquitination. A third possibility is that acetylation of Cdc25A following DNA damage represents a mechanism to ensure that Cdc25A is not entirely degraded and that some Cdc25A is quickly available following recovery.

Disruption of Cdc25A function during tumor formation due to altered Cdc25A acetylation might impair the ability of cells to control cell cycle checkpoints and thereby increase genomic instability. This notion would be consistent with Cdc25A dysregulation seen in several cancer types. Alternatively, loss of function or dysregulation of ARD1 and HDAC11 that disrupt steady state Cdc25A acetylation might also explain aberrant Cdc25A levels in those cancers. Although much regarding Cdc25A acetylation remains unresolved, our data provide new mechanistic insights into the role of Cdc25A acetylation in cell cycle regulation following genomic insult and in cancer.

## MATERIALS AND METHODS

### Cell culture, transfection and treatments

Cell culture media and reagents were purchased from Invitrogen. Both HEK 293 and HEK 293T cells were cultivated in high glucose Dulbecco's modified Eagle's medium (DMEM) supplemented with 10% fetal bovine serum (FBS), 2 mM glutamine, penicillin (100 U/ml) and streptomycin (100 mg/ml) in a humidified incubator with atmosphere containing 5% CO_2_ at 37°C. Transient transfections were performed for 24 h using Turbofect reagent (Thermo Scientific) following the manufacturer's recommendations. Briefly, growth medium in cell cultures was replaced with FBS-free DMEM. TurboFect (2 μl) and DNA (0.5 μg of DNA per 10 cm^2^ of the culture plate area) were combined in 200 ml of serum free media, incubated for 30 min at room temperature and added to cell cultures. After 4 h of culture, appropriate volume of fresh FBS was added to culture medium. To generate DNA damage, cells were incubated in growth medium containing either 1 mM methyl methanesulfonate (MMS) for 4 h, 2 mM hydroxyurea (HU) for 16 h, 20 μM etoposide for 6 h, and 1 μM Sodium Arsenite for 24 h. For inhibition of deacetylases, cells were incubated in growth medium containing 10 μM Trichostatin A (TSA) (or increasing concentrations from 0.01-20 μM when indicated), 5 mM Nicotinamide, and/or 10 mM Sodium Butyrate for 4 h. The DNA damaging agents and deacetylase inhibitors were purchased from Sigma-Aldrich. Cycloheximide (CHX, Sigma) concentration was 10 mg/ml.

### Proteins purification

FLAG-Cdc25A was purified by transfecting FLAG-Cdc25A plasmids (8 μg) into HEK 293T cells. Cells were harvested 24 h after transfection and lysed in lysis buffer (50 mM Tris-HCl, pH 7.4, 150 mM NaCl, 1 mM EDTA, 1% Triton X-100). Cells extract were incubated overnight at 4°C with Anti-FLAG M2 beads (Sigma). The beads were washed three times with TBS (50 mM Tris-HCl, pH 7.5, 150 mM NaCl) and the bound proteins were eluted using FLAG-Peptide (Sigma) in FLAG-elution buffer (50 mM Tris-HCl, pH 7.4, 0.1% NP40, 100 mM NaCl, 10% Glycerol, 5 mM DTT) for 2 h at 4°C. After elution the protein were resolved in 8% SDS-PAGE and analyzed by Western Blot with anti-FLAG (Sigma) and anti-Cdc25A (Santa Cruz) antibodies. Protein concentrations were determined using a Nanodrop (Thermo Scientific).

GFP-ARD1 was recovered by transfecting GFP-ARD1 plasmids (8 ug) into HEK 293T cells. Cells were harvested and lysed 24 h after transfection, using sonication in lysis buffer (see above). Cells extract were incubated overnight at 4°C with anti-GFP-Trap beads (Antibodies Online). Then, the beads were washed two times with TBS. The bound proteins were eluted using 0.1 M glycine (pH 2.5) followed by neutralization with 1 M Tris-Base (pH 10.4). After elution, glycereol (10% Glycerol) and DTT (5 mM) were added to the eluted proteins. To confirm that the system works the eluted protein products were resolved by 12% SDS-PAGE and analyzed by western blot with anti-GFP (Jackson ImmunoResearch) or anti- ARD1 (Santa Cruz Biotechnology) antibodies. Proteins concentrations were determined using a Nanodrop.

His-HDAC11, HA-Ubiquitin, and His-Cdc25A were purchased from Enzo Life Sciences. BSA was purchased from New England Biolabs. Myc-DDK-ARD1 was purchased from Origene.

### Co-immunoprecipitation experiments

For studies of endogenous proteins HEK 293T cells were lysed by sonication in RIPA buffer (50 mM Tris-HCl (pH 7.4), 150 mM NaCl, 1% NP-40, 0.25% sodium deoxycholate, and 1 mM EDTA) supplemented with protease inhibitor cocktail (Sigma-Aldrich), 1 mM PMSF (Sigma), and 10 units/ml of DNase I (New England Biolabs). After centrifugation at 21,000 × g for 12 min at 4°C, supernatants were removed and their protein concentrations measured. Samples (500 μg of protein each) were pre-cleared with equilibrated Protein G Plus/Protein A agarose beads (Santa Cruz Biotechnology) and 1 μg of normal IgG (Santa Cruz Biotechnology) for 1 h, at 4°C, then incubated with the corresponding antibody (anti-Cdc25A, anti-ARD1, and anti-HDAC11 from Santa Cruz Biotechnology) and 30 μl of Protein G Plus/Protein A bead suspension for 18 h at 4°C. After collection by centrifugation and removal of supernatant, the beads were then washed three times with RIPA buffer supplemented with protease cocktail inhibitors, 1 mM PMSF and 200 μg/ml ethidium bromide. After the final wash, equal portions of RIPA and 2 × SDS sample buffer (4% SDS, 20% glycerol, 0.05% bromophenol blue, and 2 M 2-mercaptoethanol) were added to the beads and immunoprecipitated proteins were released by heating at 90°C for 5 min. Equal volumes of each sample were resolved by SDS-PAGE (10%). For loading control, 50 ng of each clarified lysate was resolved by SDS-PAGE (10%) and proteins were transferred to PVDF membranes (Bio-Rad) by electroblotting. The membranes were blocked with 5% nonfat dry milk in TBST buffer (20 mM Tris, pH 7.4, 150 mM NaCl and 0.1% Tween-20) and analyzed by Western analysis with anti-Cdc25A (Santa Cruz), anti-ARD1 (Santa Cruz Biotechnology), anti-HDAC11 (Santa Cruz Biotechnology), or with anti-β-actin antibody (Sigma) for 18 h at 4°C followed by chemiluminescent detection using ECL Plus (Thermo Scientific).

For *in vitro* studies the recombinant purified proteins were incubated in RIPA buffer. After immunoprecipitation, using anti-FLAG M2 beads or anti-GFP-Trap beads, the immunoprecipitated products were collected (washed and treated as above) and subjected to SDS-PAGE and western blotting with anti-GFP (Jackson ImmunoResearch) and anti-FLAG antibodies (Agilent Technologies).

### Far western assays

Purified FLAG-Cdc25A, GFP-ARD1, His-HDAC11, and corresponding concentrations of BSA (as control) were applied directly onto nitrocellulose membranes (at the concentrations indicated in figures). The samples were dried for 15 min at 4°C. The membranes were blocked for 1.5 h at 4°C with 5% nonfat dry milk in TBST, followed by incubation with 5% milk solution containing purified FLAG-Cdc25A, GFP-ARD1, or His-HDAC11 for 3 h at 4°C. Membranes were washed 3 times, for 10 min each, with TBST, and were subjected to immunodetection with mouse anti-Cdc25A (Santa Cruz Biotechnology), mouse anti-GFP (Jackson ImmunoResearch), mouse anti-FLAG (Agilent Technologies), or mouse anti-His (Oncogene) antibodies for 1 h at 4°C (see figure legends for specifics of each experiment). The incubation with the appropriate HRP-linked secondary antibodies (Santa Cruz Biotechnology) was for 1 h at room temperature. Signal was detected using ECL Plus and visualized by autoradiography.

### *In vitro* acetylation assay

Purified FLAG-Cdc25A was incubated for 2 h at 4°C or 37°C in 20 ul of acetylation buffer (1 mM DTT, 10 mM Na-Butyrate, 0.1 mM EDTA, 10% Glycerol, 50 mM Tris (pH 8.0), 1 mM PMSF, 20 mM MgCl_2_, 1 mM ZnCl_2_, 500 mM NaCl, 0.01 mM Acetyl CoA) in the presence or absence or GFP-ARD1. Reactions were stopped by addition of 2 × SDS sample buffer. Samples were heated at 90°C for 5 min and equal volumes of each sample were resolved by SDS-PAGE (10%). Proteins were transferred to PVDF membranes by electroblotting and analyzed by Western Blot analysis with anti-acetylated lysine, anti-Cdc25A, or anti-ARD1 (all antibodies were from Santa Cruz Biotechnology) antibodies for 18 h at 4°C followed by chemiluminescent detection using ECL Plus.

### Detection of Cdc25A acetylation in cells

HEK 293T cells were lysed by sonication in RIPA buffer supplemented with protease inhibitor cocktail, 1 mM PMSF, and 10 units/ml of DNase I. After centrifugation at 21,000 × g for 12 min at 4°C, supernatants were isolated and their protein concentrations measured. Samples (500 μg of protein each) were pre-cleared with equilibrated Protein G Plus/Protein A agarose beads and 1 μg of normal mouse IgG for 1 h, at 4°C, then incubated with anti-Cdc25A (Thermo Scientific) and 30 μl of Protein G Plus/Protein A bead suspension for 18 h at 4°C. After collection by centrifugation and removal of supernatant, the beads were then washed three times with RIPA buffer. After the final wash, equal portions of RIPA and 2 × SDS sample buffer were added to the beads and immunoprecipitated proteins were released by heating at 90°C for 5 min. Equal volumes of each sample were resolved by SDS-PAGE (10%). For loading control, 50 ng of each clarified lysate was resolved by SDS-PAGE (10%). Proteins were transferred to PVDF membranes by electroblotting and analyzed by Western analysis with anti-acetylated lysine antibody (Santa Cruz Biotechnology) for 18 h at 4°C followed by chemiluminescent detection using ECL Plus. The same procedure was used for detection of Cdc25A acetylation after ARD1 overexpression in HEK 293T cells (see transfection procedures for details).

### RT-PCR

Total RNAs were extracted from cells followed by reverse transcription using Sensiscript RT kit (Qiagen), following the recommended protocol. PCR was performed using primers reported in supplementary material figure S2. The expression level of Cdc25A was calculated as a ratio of the mRNA level relative to the mRNA level for glyceraldehyde-3-phosphate dehydrogenase (GAPDH) in the same cDNA. Data represents average values of three independent experiments.

### Detection of endogenous cell cycle regulators

Cells were lysed by sonication in RIPA buffer and the protein concentration was determined using a Nanodrop. Equal protein amounts were resolved by SDS-PAGE (10%). Proteins were transferred to PVDF membranes (Bio-Rad) by electroblotting. The membranes were blocked with 5% nonfat dry milk in TBST buffer and analyzed by Western analysis with anti-Flag (Sigma), anti-Cdc25A (Thermo Scientific), and/or anti-β-actin (Sigma). Anti-Cdc25B, anti-Cdc25C, anti-cyclin A2, anti-cyclin E1, anti-cyclin D1, anti-cyclin D3, and anti-pRB were purchased from Santa Cruz Biotechnology.

### Ubiquitination assay

Purified His-Cdc25A and HA-Ub proteins were incubated in 100-ul reaction mixture containing 8 μl 5 X ubiquitination buffer (100 mM tris-HCl, pH 7.4, 25 mM MgCl2, 2.5 mM DTT, 10 mM ATP), 100 μg of HEK 293T cell lysates +/–Myc-DDK-ARD1 for 1 h at 37°C. The reaction mixture was then incubated with anti-HA antibody (Santa Cruz Biotechnology). The proteins were collected on protein A/G beads, washed with RIPA buffer supplemented with protease cocktail inhibitors, 1 mM PMSF and10 units/ml of DNase I. After the final wash, equal portions of RIPA and 2 × SDS sample buffer were added to the beads and immunoprecipitated proteins were released by heating at 90°C for 5 min. Equal volumes of each sample were resolved by SDS-PAGE (10%). Proteins were transferred to PVDF membranes by electroblotting. The membranes were blocked with 5% nonfat dry milk in TBST buffer and analyzed by Western analysis with anti-Cdc25A (Santa Cruz) for 18 h at 4°C followed by chemiluminescent detection using ECL Plus.

### Immunofluorescence

Cells cultivated on glass coverslips (transfections performed as explained above) were fixed using 2% paraformaldehyde in PBS. Following three washes with 1X PBS, coverslips were incubated 1 hour at RT with 1% BSA and 0.1% Tween 20 in PBS (blocking solution). Following overnight incubation with anti-Cdc25A antibody (1:250) in blocking solution at 4°C, coverslips were washed three times with PBS and incubated with a secondary antibody (Alexa Fluor 546, Invitrogen, Eugene, OR, USA) solution in PBS for 1 hour at RT. Incubation solution was replaced for 15 minutes with DAPI (0.1 mg/ml) dissolved in PBS. After three washes, coverslips were mounted on microscope slides and examined using Zeiss Axioplan Imaging 2 fluorescence microscope (Carl Zeiss Microimaging, Thornwood, NY) equipped with Orca ER CCD camera (Hamamatsu, Bridgewater, NJ).

### Phosphatase assay

The phosphatase activity of Cdc25A was measured using a spectrophotometric assay (EnzChek Phosphatase assay kit, Molecular Probes) following the manufacturer instructions. Shortly, the assay is based on the ability of Cdc25A to catalyze the hydrolysis of 6, 8-difluoro-4-methylumbelliferyl phosphate (DiFMUP) to 6, 8-difluoro-4-methylumbelliferone, a chromogenic product. Recombinant His-Cdc25A was incubated with substrate in the absence or presence of recombinant Myc-DDK-ARD1. Cdc25A activity was assayed at 10 minute intervals during one hour. Cdc25A activity was calculated by measuring the absorbance of the substrate at 410 nm and subtracting the control background value.

### Flow cytometry analysis

To determine the cell cycle profile of HEK 293 cells after ARD1 overexpression (see transfection protocol above for details), HEK 293 cells were cultivated to ∼50% confluency and transfected with either GFP or GFP-ARD1 constructs. After 24 h transfection, cells were trypsinized, washed twice in PBS, and fixed overnight in cold 0.25% paraformaldehyde in PBS. Then, cells were resuspended in 70% ethanol at −20°C (added drop-wise to the cell pellet with the tube sitting on a vortex) and stored at 4°C until analyzed. Fixed cells were then washed with PBS and stained with solution of 10 μg/ml propidium iodide (Molecular Probes) and 40 μg/ml RNase A (Sigma) in PBS for 30 min in the dark at 37°C. Samples were filtered through a nylon mesh to remove clumps before acquisition on a BD LSR II flow cytometer system (Becton Dickinson).

### Deacetylation assays

For the *in vitro* deacetylation assay purified FLAG-Cdc25A was incubated for 2 h at RT in 20 μl of deacetylation buffer (1 mM DTT, 0.1 mM EDTA, 10% Glycerol, 50 mM Tris (pH 8.0), 1 mM PMSF, 20 mM MgCl_2_, 1 mM ZnCl_2_, 500 mM NaCl) containing increasing amount of recombinant His-HDAC11. Reactions were stopped by addition of 2 × SDS sample buffer. Samples were heated at 90°C for 5 min and equal volumes of each sample were resolved by SDS-PAGE (10%). Proteins were transferred to PVDF membranes by electroblotting. The membranes were blocked with 5% nonfat dry milk in TBST buffer and analyzed by Western analysis with anti-acetylated lysine (Santa Cruz Biotechnology) or anti-Cdc25A (Thermo Scientific) antibody for 18 h at 4°C followed by chemiluminescent detection using ECL Plus.

To determine if HDAC11 deacetylates endogenous Cdc25A, HEK 293T cells were lysed by sonication in RIPA buffer supplemented with protease inhibitor cocktail, 1 mM PMSF, and 10 units/ml of DNase I. After centrifugation at 21,000 × g for 12 min at 4°C, supernatants were isolated and their protein concentrations measured. Samples (500 μg of protein each) were pre-cleared with equilibrated Protein G Plus/Protein A agarose beads and 1 μg of normal rabbit IgG (Santa Cruz Biotechnology) for 1 h, at 4°C, then incubated with rabbit ant-Cdc25A antibody (Thermo Scientific) and 30 μl of Protein G Plus/Protein A bead suspension for 18 h at 4°C. After collection by centrifugation and removal of supernatant, the beads were then washed three times at 4°C for 5 min with a deacetylation buffer. After the final wash, 25 μl of deacetylation buffer was added to the beads and 2.5 μg of purified His-HDAC11 was added to the corresponding reaction. The reactions were incubated 2 h at RT. Reactions were stopped by addition of 2 × SDS sample buffer. Samples were heated at 90°C for 5 min and equal volumes of each sample were resolved by SDS-PAGE (10%). For loading control, 25 ng of each clarified lysate was resolved by SDS-PAGE (10%). Proteins were transferred to PVDF membranes by electroblotting. The membranes were blocked with 5% nonfat dry milk in TBST buffer and analyzed by Western analysis with anti-acetylated lysine (Santa Cruz Biotechnology) or anti-actin (Sigma) antibody for 18 h at 4°C followed by chemiluminescent detection using ECL Plus.

### Statistical analysis

Comparative differences for Cdc25A acetylation after DNA damaging agents or inhibition of deacetylases were analyzed using one-way ANOVA (GraphPad Prism-5). Cdc25A acetylation was considered significantly increased between control and the different treatments if an effect was observed at *p* < 0.05. The analysis was followed by Newman Keuls post-test.

## SUPPLEMENTARY MATERIALS FIGURES


