# Supplementary material for: Acetylation and deacetylation of Cdc25A constitutes a novel mechanism for modulating Cdc25A functions with implications for cancer
Source: Oncotarget. 2016 Mar 7;7(15):20425–39. doi: 10.18632/oncotarget.7966 (PMC4991465; doi:10.18632/oncotarget.7966)
Supplement: Supplementary file 1 [file oncotarget-07-20425-s001.pdf]

Acetylation and deacetylation of Cdc25A constitutes a novel mechanism for modulating Cdc25A functions with implications for cancer

Supplementary Materials

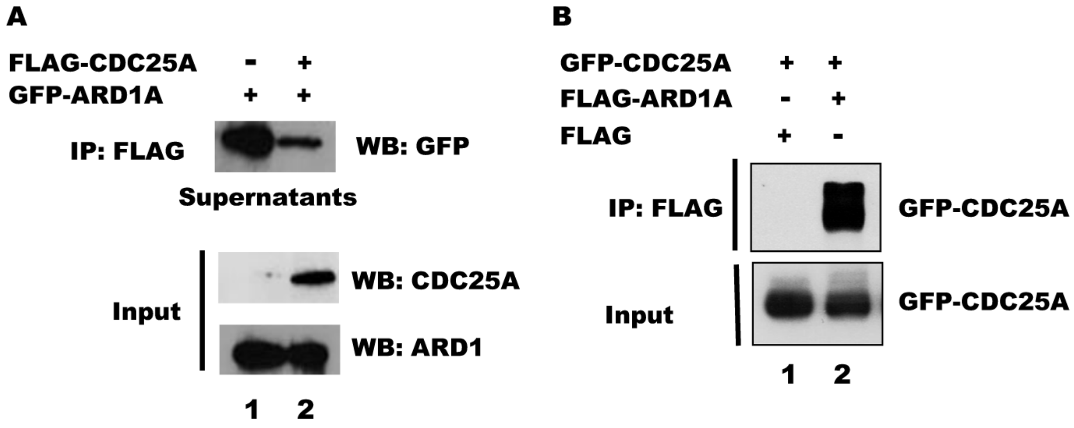

Supplementary Figure S1: Cdc25A and ARD1 interact *in vitro* and in cell lysates. (**A**) ARD1A co-immunoprecipitates with Cdc25A *in vitro*. Purified GFP-ARD1A alone (lane 1) or with FLAG-Cdc25A (lane 2) was incubated with anti-FLAG M2 beads. After immunoprecipitation, the supernatants of the reactions were analyzed for GFP-ARD1A immunodepletion by SDS-PAGE (10%) and Western blotting using anti-GFP antibody. (**B**) ARD1 and Cdc25A associate in a co-transfection experiment. Cells were co-transfected with GFP-Cdc25A and either FLAG vector (lane1) or FLAG-ARD1A (lane 2). After immunoprecipitation with anti-FLAG-M2 beads, the immunoprecipitates were analyzed using anti-GFP antibody, followed by chemiluminescent detection.

RT-PCR primers:

hARD1A	GAGGTGATATGTCCATGGGG	CCCCAGCTCTCTTACATTGC
hcdc25a	ACATTTGGTTGCTGGGTCTT	AGGTCTCCTCATGACAAGGG
hGAPDH	GAA GGTGAAGGTCGGAGTC	GAAGATGGTGATGGGATTTC

siRNA targets::

ARD1A/B siRNA target sequence 1	AACTACCAGATGAAATACTAC
ARD1A/B siRNA target sequence 2	AACACCCTCAACTTTCAGATC

Supplementary Figure S2: Primers for RT-PCR for hARD1A, Cdc25A and GAPDH, and two siRNA oligonucleotide sequences for silencing ARD1A/B.

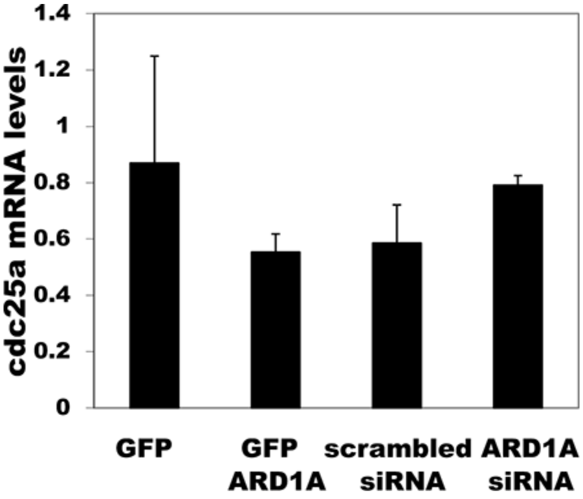

Supplementary Figure S3: ARD1 levels do not affect Cdc25A mRNA levels. Levels of Cdc25A mRNA in cells transfected with GFP-ARD1A or with siRNA targeting ARD1A/B were analyzed by RT-PCR. Bar graph represents average values from three independent experiments.

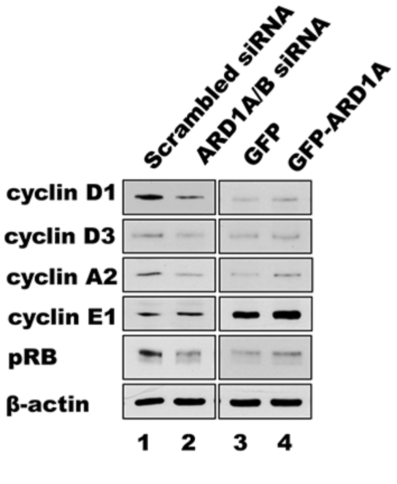

Supplementary Figure S4: Effect of ARD1 levels on cell cycle regulatory proteins. The levels of cyclin D1, cyclin D3, cyclin A2, cyclin E1 and pRB were measured by Western blot after HEK 293T cells were transfected with a control scrambled siRNA (lane 1) or ARD1A/B siRNAs (lane 2). For overexpression of ARD1, plasmids expressing GFP (control, lane 3) or GFP-ARD1A lane 4) were transfected into HEK 293T cells. After 24 h cell lysates were analyzed by Western blotting using antibodies specific for the same cell cycle proteins.

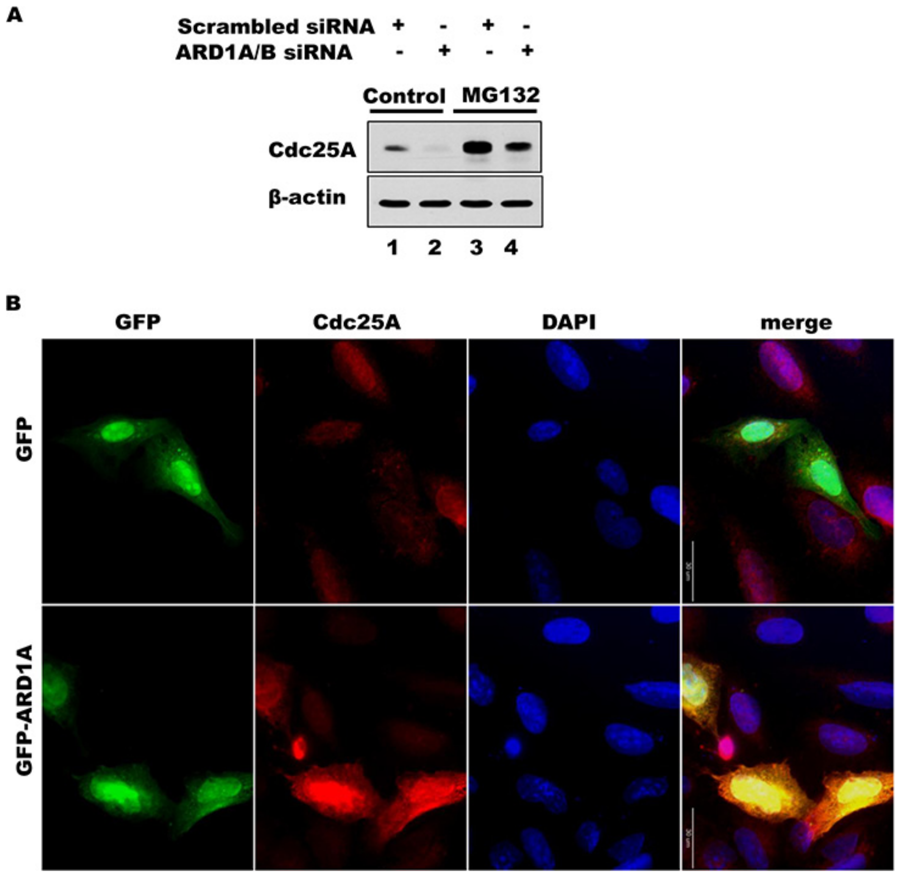

Supplementary Figure S5: ARD1 expression modulates Cdc25A abundance. (**A**) Inhibition of proteasome function by MG132 does not prevent the total decrease of Cdc25A expression induced by ARD1A/B siRNA. Cells were left untreated (lanes 1 and 2) or were treated with 10 µM MG132 for 24 hours (lanes 3 and 4) either with (lanes 2 and 4) or without (lanes 1 and 3) ARD1A/B siRNA. The level of Cdc25A in each case was assessed by Western blot. (**B**) ARD1 overexpression increases the abundance of Cdc25A. Cells were transfected with either GFP (upper panels) or GFP-ARD1A (lower panels) and Cdc25A was detected by immunocytochemistry. Nuclei were stained with DAPI (blue). Co-localization is indicated in the yellow in the merged views.

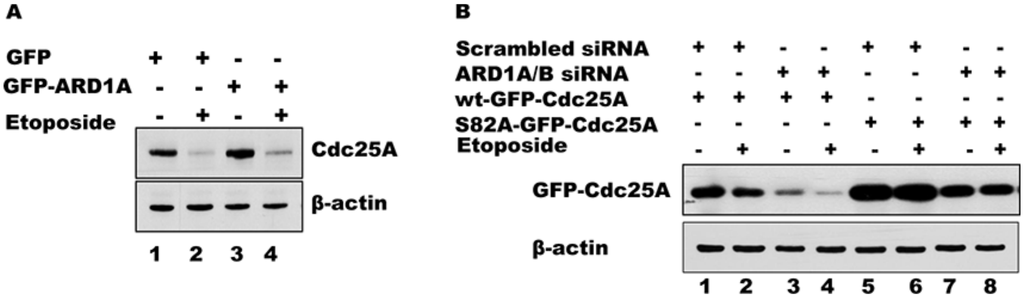

Supplementary Figure S6: Downregulation of endogenous Cdc25A induced by etoposide is not prevented by GFP-ARD1A expression. (**A**) Cells transfected with GFP (lanes 1 and 2) or with GFP-ARD1 (lanes 3 and 4) were left untreated (lanes 1 and 3) or were treated with 40 μM etoposide for 24 hours to generate DNA damage (lanes 2 and 4). The level of Cdc25A protein was assessed by Western blot in each case. (**B**) ARD1A/B siRNA reduces the level of S82A-Cdc25A-GFP mutant independent of DNA damage. HEK 293T cells were transfected with a scrambled siRNA (lanes 1,2, 5, and 6) or an siRNA against ARD1A/B (lanes 3, 4, 7, and 8) to establish the efficacy of ARD1 knockdown. Cells with wildtype Cdc25A treated with 40 μM etoposide (lane 2) for 24 hours showed a reduced level of Cdc25A compared with the control. When co-transfected with ARD1A/B siRNA, the level of Cdc25A was further decreased (lane 4). Cells transfected with control siRNA and S82A-Cdc25A-GFP mutant (lanes 5 and 6) were refractory to etoposide-induced degradation (lane 6). Cells transfected with ARD1A/B siRNA (lanes 7 and 8) had a reduced level of mutant Cdc25A compared to cells transfected with control siRNA (lanes 5 and 6), but there was no further reduction in Cdc25A abundance with etoposide treatment (lane 8) compared to untreated cells (lane 7).
